# Aryl hydrocarbon receptor (AHR) functions in infectious and sterile inflammation and NAD^+^-dependent metabolic adaptation

**DOI:** 10.1007/s00204-021-03134-9

**Published:** 2021-09-24

**Authors:** Karl Walter Bock

**Affiliations:** Institute of Experimental and Clinical Pharmacology and Toxicology, Wilhelmstrasse 56, 72074 Tübingen, Germany

**Keywords:** AHR, CD38, PARP7, Sirtuins, Infectious and sterile inflammation

## Abstract

Aryl hydrocarbon receptor (AHR) research has shifted from exploring dioxin toxicity to elucidation of various physiologic AHR functions. Exposure to 2,3,7,8-tetrachlorodibenzo-p-dioxin (TCDD) is known to exert cellular stress-mediated sterile inflammatory responses in exposed human tissues but may be lethal in sensitive species. Inflammation can be thought of as the extreme end of a spectrum ranging from homeostasis to stress responses (sterile inflammation) and to defense against infection (infectious inflammation). Defense against bacterial infection by generation of reactive oxygen species has to be strictly controlled and may use up a considerable amount of energy. NAD^+^-mediated energy metabolism adapts to various inflammatory responses. As examples, the present commentary tries to integrate responses of AHR and NAD^+^-consuming enzymes (PARP7/TiPARP, CD38 and sirtuins) into infectious and stress-induced inflammatory responses, the latter exemplified by nonalcoholic fatty liver disease (NAFLD). TCDD toxicity models in sensitive species provide hints to molecular AHR targets of energy metabolism including gluconeogenesis and glycolysis. AHR research remains challenging and promising.

## Introduction

Aryl hydrocarbon receptor (AHR) research has shifted from exploring dioxin toxicity to elucidation of various physiologic AHR functions. AHR has been characterized as ligand-activated, multifunctional transcription factor and environmental sensor (Gu et al. 2000; Avilla et al. 2020). Accumulating evidence suggests that AHR, mainly expressed in barrier organs such as skin, intestine and lung, is involved in multiple physiologic functions including development (Gasiewicz et al. 2014; Ko et al. 2016), chemical defense (Nebert et al. 2004), microbial defense (Lawrence and Vorderstrasse 2013), immunity and inflammation (Stockinger et al. 2014; Esser and Rannug 2015), reproduction (Baba et al. 2005), and energy metabolism (Diani-Moore et al. 2017).

Exposure to 2,3,7,8-tetrachlorodibenzo-p-dioxin (TCDD) is known to exert sterile inflammatory responses in exposed tissues (Saurat et al. 2012). Classic causes of inflammation (infection and tissue injury) may be one end of a range of adverse conditions starting from tissue homeostasis to tissue stress-mediated sterile inflammation, and ending with infectious inflammation by pathogens (Medzhitov 2008; Wang et al. 2019). In infectious inflammation the innate immune system is activated by recognizing, e.g., pathogenic bacteria through pattern-recognition receptors (PRRs). PRRs such as TLRs (Toll-like receptors) are expressed by many cell types such as epithelial cells of barrier organs and associated immune cells including neutrophils, macrophages, and dendritic cells. PRRs recognize pathogen-associated molecular patterns (PAMPs). In the case of sterile inflammation, danger-associated molecular patterns (DAMPs) are similarly recognized by a variety of receptors and other factors triggered, e.g., by structures of dying cells.

Since inflammatory responses are energy-consuming processes they have to adapt to NAD^+^-dependent energy economy of the organism (Medzhitov 2008; Wang et al. 2019). NAD^+^ is an essential cofactor for redox enzymes and a substrate for signaling enzymes. Energy consumption by various inflammatory responses is regulated by NAD synthesis and consumption. NAD^+^-consumption is carried out by multiple enzymes such as CD38 (Verdin 2015; Yang and Sauve 2016), 17 PARPs including the AHR target gene PARP7/TiPARP (MacPherson et al. 2013; Gupte et al. 2018) and 7 NAD-dependent sirtuins (Chang and Guarente 2014; Singh et al. 2018).

Elucidation of AHR functions is challenged by dependence upon cell type, cellular context, and species differences, the latter suggested by TCDD toxicity in sensitive species leading to wasting syndrome and lethality (Poland and Knutson 1982). Therefore, the commentary is focused to inflammatory responses in intestine, liver and associated immune cells in humans. After an overview on AHR and NAD^+^-consuming enzymes (PARP7/TiPARP, CD38 and sirtuins) the commentary tries to integrate functions of AHR and NAD^+^-consuming enzymes in infectious and sterile inflammatory responses. TCDD toxicity models in sensitive species are discussed since they provide hints to molecular AHR targets in energy metabolism.

## AHR ligands and signaling

A large number of AHR ligands has been identified and comprehensively reviewed (Denison and Nagy 2003; Nguyen and Bradfield 2008; Esser and Rannug 2015; Murray and Perdew 2017; Rothhammer and Quintana 2019). Prototypical AHR agonist TCDD is the most potent but poorly metabolized ligand leading to persistent AHR activation and dysregulation of AHR functions. 6-Formylindolo[3,2-b]carbazole (FICZ) is an equally potent AHR ligand that is rapidly metabolized by the AHR target CYP1A1 leading to an important negative feedback loop (Esser and Rannug 2015). Relevance of the AHR-CYP1-FICZ axis in vivo has been demonstrated by overexpression of CYP1A1 (Schiering et al. 2017). Notably, toxicity of TCDD and dioxin-like compounds leading to potent and persistent AHR activation has to be distinguished from that of polycyclic aromatic hydrocarbons (PAHs) such as benzo[a]pyrene that, e.g., do not lead to chloracne, the hallmark of dioxin toxicity (Avilla et al. 2020). Recently, vitamin B12 and folic acid have been identified as endogenous AHR antagonists (Kim et al. 2020).

There are diverse sources of AHR agonists and antagonists (Table 1). Dietary phytochemicals and microbial ligands represent natural AHR ligands since they have been characterized as physiological agents necessary for intestinal development (Kiss et al. 2011) and for homeostasis with commensal bacteria (Lamas et al. 2018). Many fruits and herbs contain AHR agonistic flavonoids such as quercetin and glucobrassicin, the latter generating the pro-ligand indole-3-carbinol that is further converted to the strong AHR ligand ICZ (indolo[3,2-b]carbazole). Dietary flavonoids such as quercetin belong to many indirect AHR agonists that inhibit CYP1A1 and thereby may increase the endogenous ligand FICZ. Microbiota such as Pseudomonas aeruginosa and mycobacterium tuberculosis have been demonstrated to generate AHR agonistic virulence factors such as pyocyanin, 1-hydroxyphenazine and phthiocol (Moura-Alves et al. 2014, 2019). Similar microbiota-generated virulence factors may be generated in the intestine. When recognized by AHR expressed in intestinal immune cells such as ILC3, neutrophils are recruited that generate the oxidative burst to kill pathogenic bacteria (Bock 2020a, for references).

**Table 1:** Selected agonists and antagonists of AHR

Sources of ligands	Agonists and antagonists^b^ of AHR
Endogenous chemicals	Indole-3-acetaldehyde^a^
	FICZ
	Kynurenine
	Bilirubin
	Lipoxin A
	Prostaglandin G2
	Vitamin B12^b^
	Folic acid^b^
Phytochemicals	Indole-3-carbinol^a^
	Indole-3-carboxaldehyde^a^
	ICZ
	Indigo
	Quercetin^c^
Microbial products	Indole-3-acetaldehyde^a^
	1-Hydroxyphenazine
	Pyocyanin
	Phthiocol
Drugs	StemReginin1^b^
	Omeprazole^c^
Xenobiotic chemicals	TCDD
	Benzo[a]pyrene
	3-Methylcholanthrene
	ß-Naphthoflavone

The list is by no means complete. Pro-ligands are converted to high-affinity ligands; for example, indole-3-carbinol to ICZ. Indirect agonists may inhibit CYP1A1 and thereby activate the endogenous ligand FICZ

^a^Pro-ligands

^b^antagonists

^c^Indirect agonists

AHR is also operating by non-genomic signaling including protein kinases (Puga et al. 2009). For example, it operates as component of E3 ubiquitin ligases (Ohtake et al. 2009) and of a signalsome (Bunaciu et al. 2019). Furthermore, AHR often operates in cross-talk with other transcription factors including Nrf2, the key protector against oxidative stress (Kensler et al.^2007^) and NF-κB (Vogel et al. 2014). Interestingly, multiple mechanisms evolved to avoid sustained AHR activation including cross-talk with AHR repressor (Karchner et al. 2009), the discussed AHR-CYP1A1-FICZ axis (Esser and Rannug 2015), and nuclear-cytoplasmic shuttling (Ikuta et al. 2009).

Non-genomic AHR-mediated inflammatory signaling involving tissue-specific protein kinases has been substantiated in many cells/tissues from several species (Bock 2020b, for references). Presumably, AHR is activated in both genomic and non-genomic signaling by ligands or other cues including phosphorylation. Interestingly, non-genomic AHR signaling could be demonstrated in human platelets, an anucleated cell-mediating immunity, inflammation and thrombosis (Pombo et al. 2015). In this study, 1 nM TCDD and 10 nM omeprazole significantly increased AHR protein. AHR signaling was demonstrated by increased p38-MAPK and cPLA2 phosphorylation. Non-genomic pathways leading to TCDD-mediated tissue inflammation have been discussed before including membrane translocation and activation of c-Src. These transient responses are stabilized by genomic AHR signaling including IL-6 expression (Puga et al. 1997; Matsumura 2009).

## NAD^+^ consuming enzymes (CD38, PARPs, and NAD^+^-dependent sirtuins)

**Fig. 1:**
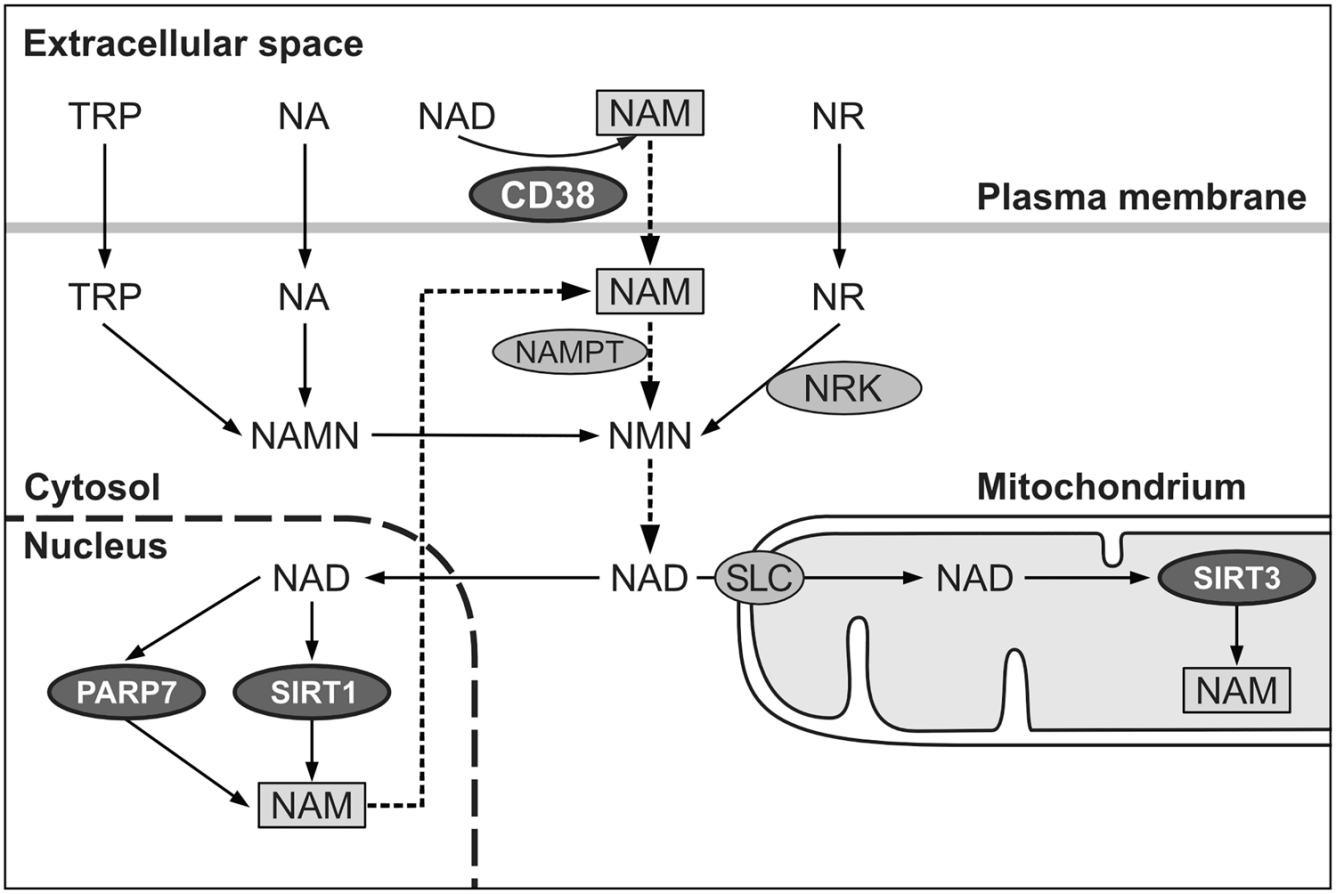
Simplified scheme of NAD^+^-consuming enzymes (CD38, PARPs and sirtuins), and reutilisation of generated nicotinamide (NAM) by the salvage pathway of NAD synthesis (dashed arrows). TRP, tryptophan; NA, nicotinic acid; NR, nicotinamide riboside; NRK, nicotinamide riboside kinase; NMN, nicotinamide mononucleotide; SLC, mitochondrial NAD^+^ transporter (Luongo et al. 2020). In the salvage pathway of NAD synthesis, NAM generated by all NAD-consuming enzymes is reutilized via the rate-limiting enzyme NAMPT (nicotinamide phosphoribosyltransferase) to synthesize NMN

As mentioned before, inflammatory responses may be energy-consuming processes and have to be metabolically adapted (Medzhitov 2008; Wang et al. 2019). Adaptation is achieved in part by NAD^+^ homeostasis. In addition to NAD synthesis, accumulating evidence suggests that NAD homeostasis is achieved by NAD-consuming enzymes including CD38, PARPs and sirtuins (Canto et al. 2015). Studies with CD38-deficient mice identified CD38 as NAD^+^-consuming enzyme involved in NAD^+^ homeostasis (Verdin 2015; Yang and Sauve 2017; Hogan et al. 2019). In addition, PARPs, particularly PARP1 and PARP7/TiPARP, are known NAD-consuming enzymes (Gupte et al. 2018; Cohen 2020; Fehr et al. 2021). Sirtuins have been identified as NAD-dependent protein deacetylases that consume a considerable amount of NAD^+^. The extent of NAD^+^ consumption by these enzymes under homeostatic or infectious stress conditions is presently unknown. It has been suggested that nicotinamide released by all NAD-consuming enzymes has to be almost completely reutilized to maintain NAD^+^ levels in nuclei, cytosol and mitochondria by the salvage pathway of NAD synthesis (Fig. 1). Estimates suggest that the entire NAD pool is replaced 2–4 times per day, and that only 0.1–0.2% nicotinamide is lost per turnover cycle (Yang and Sauve 2017). De novo NAD synthesis from tryptophan (TRP) is not sufficient to maintain NAD^+^ levels, particularly in stressed conditions such as inflammatory responses. Subcellular NAD^+^ pools are compartmentalized. For example, the mitochondrial NAD^+^ pool is distinct from the cytosolic pool. There has been much debate how mitochondrial NAD is generated. Recently, a mitochondrial NAD carrier has been identified (Luongo et al. 2020).

The following brief overview of CD38, PARPs and sirtuins is focused on subsequently discussed interaction of AHR and NAD^+^-consuming enzymes in infectious diseases and energy metabolism.

### CD38: subcellular locations and functions

CD38 hydrolyzes NAD^+^ at the energy-rich nicotinamide-ribose bond to ADP-ribose and nicotinamide. The enzyme catalyzes multiple reactions and is involved in many signaling pathways. In addition to generating extracellular adenosine (Horenstein et al. 2019), CD38 is a cyclic ADP-ribose synthase, mobilizing calcium for important signaling reactions from endoplasmic reticulum (ER) stores. Cyclic ADP-ribose-mediated calcium signaling is involved in many important processes, e.g., secretion of insulin and oxytocin (Jin et al. 2007; Lee and Zhao 2019). Loss of CD38 renders mice susceptible to bacterial infections due to loss of chemotaxis of neutrophils. This process has been shown to depend on calcium mobilization mediated by cyclic ADP-ribose (Partida-Sanchez et al. 2001). Interestingly, CD38 together with AHR is a constituent of a signalsome (Bunaciu et al. 2019), probably regulating microbial defense.

Early cell fractionation studies of rat liver suggested that, in addition to its role as plasma membrane-bound ecto-enzyme, NAD-glycohydrolase is also a constituent of the endoplasmic reticulum (Bock et al. 1971). NAD-glycohydrolase and CD38 have been demonstrated to be identical proteins (Bertellier et al. 1998; Cockayne et al. 1998). The enigma how the ecto-enzyme CD38 could use NAD to produce intracellular cyclic ADP-ribose has finally been solved by demonstrating that CD38 can be inserted into membranes in both type II and III orientation (Lee and Zhao 2019).

### PARPs in NAD^+^ consumption

17 NAD^+^-consuming PARPs have been identified in the cell nucleus and cytoplasma (Gupte et al. 2018; Cohen 2020; Fehr et al. 2021). PARPs are NAD^+^-consuming enzymes, covalently linking a single ADP-ribose (ADPR) or a chain of ADPR units to proteins. PARP1 is the most abundant and best studied enzyme and belongs to the poly(ADP-ribosylating) proteins. It is mostly involved in DNA repair. With regard to cooperation with AHR, TCDD-induced PARP7/TiPARP (TCDD-inducible PARP) has been characterized as repressor of AHR expression (MacPherson et al. 2013) and mediator of NAD^+^-regulated dioxin toxicity (Diani-Moore et al. 2017), further discussed in the section on TCDD toxicity models in sensitive species. PARP7/TiPARP (together with PARP10, PARP12, PARP14) belongs to the mono(ADP-ribosylating) enzymes involved in sirtuin regulation (Cohen 2020).

With regard to the current COVID-19 pandemia, it should be mentioned that preliminary studies with murine hepatitis virus and human SARS-CoV-2 infection suggest increases of PARP7 and PARP10 expression and dysregulation of the NAD metabolome by decreasing de novo NAD synthesis and increasing the salvage pathway (Heer et al. 2020). However, implications of these findings are still unclear.

### NAD^+^-consuming sirtuins

Mammalian sirtuins are seven members belonging to a group of histone and protein deacetylases with different subcellular localization and function (Chang and Guarente 2014; Canto et al. 2015; Singh et al. 2018). Nuclear SIRT1 and mitochondrial SIRT3 are major NAD^+^-dependent deacetylases involved in energy, lipid and glucose metabolism. They are emerging as key regulators of energy metabolism in normal physiology and a variety of oxidative stress-mediated pathological situations. Evidence has been obtained that sirtuins regulate key rate-limiting metabolic enzymes and cofactors including AMPK (AMP-activated protein kinase) (Hou et al. 2008) and PGC1*α* (Rodgers et al. 2005). It is conceivable that in this way SIRT1 and mitochondrial SIRT3 are involved in adapting energy metabolism.

## AHR and NAD^+^-consuming enzymes in infectious inflammation of the intestine

### Homeostasis

As discussed in a previous commentary (Bock 2020a), AHR is involved in intestinal homeostasis via microbial indoles and in bacterial defense via microbial virulence factors such as pyocyanin and 1-hydroxyphenazine (Moura-Alves et al. 2014, 2019). AHR-expressing ILC3 cells are generating anti-inflammatory Il-22. They are involved in commensal-host homeostasis, and maintenance of intestinal epithelial integrity via stabilizing the tight-junction barrier (Scott et al. 2020). Evidence for an essential role for ILC3 cells has been obtained in maintaining tissue homeostasis and limiting chronic infection in the intestine of humans and mice (Qiu et al. 2013; Blander et al. 2017; Sonnenberg and Artis 2015).

### Microbial defense

**Fig. 2:**
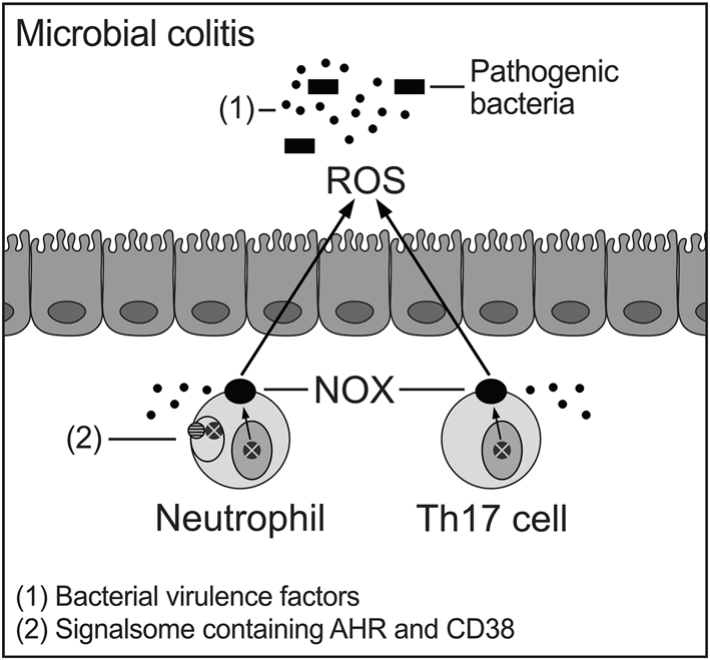
Illustration of proposed roles of AHR in inflammatory responses of bacterial colitis. Pathogenic bacteria (black rectangles) generate AHR agonistic virulence factors (1, black dots) leading to recruitment of ROS-generating Th17 cells and neutrophils, the latter expressing (i) AHR target gene p40^phox^ subunit of the NADPH oxidase complex (NOX) (Wada et al. 2013) and (ii) plasma membrane-bound signalsome (2) containing AHR (crossed circle) and CD38 (striped circle) (Bunaciu et al. 2019)

AHR-deficient mice are more susceptible to viral and bacterial diseases (Lawrence and Vorderstrasse 2013). AHR sensing of bacterial pigments alerts the host of invading pathogens. Microbial virulence factors have been identified as AHR agonists (Moura-Alves et al. 2014, 2019). AHR is involved in quorum sensing, i.e., a communication system to modify bacterial behavior through signaling molecules. In this way, intestinal epithelial cells and associated immune cells recognize these virulence factors as PAMPs, and orchestrate microbial defense recruiting neutrophils and Th17 cells that generate reactive oxygen species (ROS) to kill bacteria in the so-called respiratory burst (Esser and Rannug 2015; Medzhitov 2008; Wang et al. 2019). In addition, AHR and CD38 are constituents of a signalsome involved in neutrophil differentiation and activation (Fig. 2) (Bunaciu et al. 2019). The respiratory burst in colitis consumes a lot of energy (Wang et al. 2019). AHR has been demonstrated to regulate a subunit of the NADPH oxidase complex (NOX) (Wada et al. 2013) and to promote macrophage survival (Kimura et al. 2013). CD38 may be an integrating factor, mediating neutrophil trafficking (Partida-Sanchez et al. 2001) and possibly maintaining energy economy. It is conceivable that CD38 in the discussed signalsome may be involved in adapting energy metabolism to demands of the organism. However, underlying mechanisms are unknown.

### Resolution phase of inflammation

To prevent collateral tissue damage, ROS production has to be strictly controlled (Roy et al. 2017). In the resolution phase of inflammation, Th17 cells are reprogrammed to Treg cells, and macrophages switch to secrete anti-inflammatory cytokines (Ip et al. 2017). In addition, AHR-associated Src kinase promotes phosphorylation of indolamine-2,3-dioxygenase (IDO-1) resulting in a tolerant state (Bessede et al. 2014). Roles of NAD^+^-dependent SIRT1 as well as epigenetic and metabolic programming in endotoxin tolerance have been discussed (Liu et al. 2011; Vachharajani and McCall 2019).

## AHR and CD38 in sterile inflammation, obesity-mediated nonalcoholic fatty liver disease (NAFLD) as example

Obesity is a worldwide complex health problem. It is mainly produced by an imbalance between food intake and energy expenditure, which leads to an excessive accumulation of adipose tissue leading to dyslipidemia affecting many organs including the liver as the major organ responsible for lipid homeostasis (Jung and Choi 2014). Here, the discussion is focused on roles of AHR and CD38 in NAFLD and NASH (nonalcoholic steatohepatitis).

### Roles of AHR

**Fig. 3:**
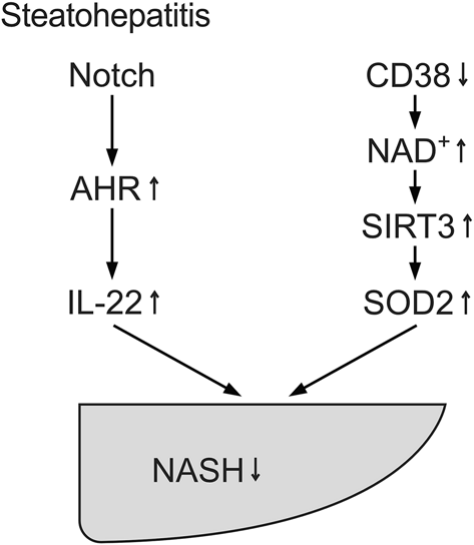
Discussed AHR- and CD38-mediated pathways attenuating steatohepatitis. AHR activation may lead to generation of anti-inflammatory IL-22. Inhibition of CD38 increases NAD^+^, leading to increased SIRT3 and SOD2 activity. SOD2 detoxifies reactive oxygen species (ROS) and thereby decreases nonalcoholic steatohepatitis (NASH). *SOD* superoxide dismutase

As discussed previously, AHR has been demonstrated to be involved in opposing processes (Bock 2020a). Sustained AHR activation by TCDD or in constitutively active AHR-expressing transgenic mice sensitizes mice to NASH (He et al. 2013). However, transient AHR activation has also been demonstrated to be involved in beneficial effects: Murine AHR activation by ß-naphthoflavone is involved in decreased lipogenesis (Alexander et al. 1998; Tanos et al. 2012). AHR activation by multiple agents and factors including quercetin and indigo has been demonstrated to prevent NASH (Yang et al. 2019; Lin et al. 2019). Activation of the Notch signaling pathway indirectly activates AHR (Alam et al. 2010; Lee et al. 2012) leading to enhanced anti-inflammatory IL-22 secretion in ILC3 and Th22 cells (Fig. 3). Effects of IL-22 have been comprehensively reviewed (Gulhane et al. 2016; Mizoguchi et al. 2018).

Notably, although beneficial effects of AHR may be achieved in isolated experimental models, sustained TCDD-activated AHR always bears the risk of facilitating chronic inflammatory responses. Interestingly, there is also a connection of fatty liver with vitamin B12 and folic acid as AHR antagonists: Mice pretreated with these vitamins prevented fatty liver produced by long-term treatment with TCDD (Kim et al. 2020).

### Roles of CD38

CD38 is necessary for high-fat diet-induced NAFLD (Barbosa et al. 2007). Hence, inhibition of CD38 may prevent NASH: Inhibition of CD38 leads to increased NAD^+^ levels (Escande et al. 2013). SIRT3 and SOD2 activity are enhanced, and oxidative stress and NASH may be attenuated (Fig. 3) (Qiu et al. 2018; Camacho-Pereira et al. 2016). Furthermore, the CD38 component of the discussed signalsome (Bunaciu et al. 2019) may contribute to the signalsome’s proposed integrating function of energy economy during inflammatory responses.

Modulation of AHR signaling and NAD^+^ homeostasis may provide therapeutic options. Transient AHR activation by multiple agents (including quercetin and indigo) may stimulate anti-inflammatory responses and prevent NASH (Yang et al. 2019; Lin et al. 2019). Similarly, dietary indol-3-carbinol-generating phytochemicals (Diaz-Diaz et al. 2016) and indole-3-acetate-generated microbial products have been demonstrated to stimulate anti-inflammatory responses (Krishnan et al. 2018; Scott et al. 2020). Recently, phytochemicals and bacterial indole derivatives including indole-3-carboxaldehyde have been suggested to improve intestinal integrity via the AHR and Il-10 by stimulating antimicrobial peptide generation and goblet cell development (Powell et al. 2020). These indole derivatives may also limit age-associated systemic inflammation. Nevertheless, as comprehensively discussed (Smirnova et al. 2016), there is no consensus about direct and indirect AHR ligands operating within particular cells and in particular contexts. In addition to AHR modulation, search for NAD-boosting molecules including nicotinamide riboside (NR) may synergistically attenuate inflammatory responses (Rajman et al. 2018). NR enhances oxidative metabolism and protects against high-fat diet-induced obesity via enhancing SIRT1 and SIRT3 activation (Canto et al. 2012). Safety and metabolism of NR has been tested in a clinical trial (Conze et al. 2019). In addition, NR depressed levels of circulating inflammatory cytokines in aged adults (Dollerup et al. 2018).

AHR is involved in IDO-kynurenine-mediated tolerance and immunosuppression (Bessede et al. 2014). In this way, AHR is involved in cancer cell-mediated immunosuppression, PD-1 up-regulation in CD8^+^ T cells, and resistance to immune checkpoint inhibitors in different IDO-1 overexpressing cancer types. In these conditions it has been demonstrated that blockade of AHR would overcome this limitation (Campesato et al. 2020). Hence, anti-inflammatory AHR activation may not be justified in cancer patients. A similar note of caution is warranted in chronic inflammatory disease leading to autoimmunity.

## TCDD toxicity models in sensitive species providing hints to molecular AHR targets of energy metabolism

TCDD toxicity is known to be species dependent leading in sensitive species to wasting syndrome and lethality (Poland and Knutson 1982). Early studies suggested that TCDD reduced gluconeogenic enzymes in liver including PEPCK (phosphoenolpyruvate carboxykinase) (Weber et al. 1991). TCDD toxicity sensitive and resistant rat strains were also identified (Viluksela et al. 1999). Targets of TCDD toxicity have been further identified in the chick embryo model: AHR-regulated PARP7/TiPARP was found to decrease NAD^+^ levels and suppress gluconeogenesis by decreasing the expression of PEPCK and its coactivator PGC1α due to decreased SIRT1-mediated ADP-ribosylation of these genes. Addition of nicotinamide, the major substrate of the NAD^+^ salvage pathway, corrected these effects (Diani-Moore et al. 2010, 2013). NAD^+^ loss was found to be attributable to increased PARP activity in thymus and liver, leading to thymus atrophy and hepatosteatosis (Diani-Moore et al. 2017). Effects of TCDD were found to be organ specific leading to decreased glycolytic enzymes and glucose transporters in thymus and increased glycolytic enzymes and glucose transporters in liver. In TCDD-treated primary cultures of human hepatocytes glycolysis and GLUT1 were also increased (Diani-Moore et al. 2020). These findings provide hints to molecular AHR targets of energy metabolism. However, many questions remain.

## Conclusions

Accumulating evidence suggests that AHR is involved in infectious and sterile inflammatory responses. These processes may use up a considerable amount of energy. Therefore, energy metabolism has to be metabolically adapted (Medzhitov 2008; Wang et al. 2019). An excess of bactericidal ROS may lead to collateral host tissue injury and has to be strictly controlled. Metabolic adaptation appears to be achieved by NAD homeostasis (Verdin 2015; Yang and Sauve 2017; Hogan et al. 2019). In addition to NAD synthesis, NAD^+^ homeostasis appears to be regulated by NAD^+^-consuming enzymes including CD38, PARPs such as PARP1, PARP7/TiPARP and sirtuins such as SIRT1 and SIRT3. Roles of AHR and NAD-consuming enzymes have been discussed using infectious inflammation (bacterial infection) and sterile inflammation (nonalcoholic fatty liver disease) as examples.

Multiple sources of endogenous, phytochemical and microbial AHR ligands have been identified allowing intense interaction of the receptor and sensor between barrier organs and the environment. Connections between AHR and NAD^+^-consuming enzymes (PARP7/TiPARP, CD38 and sirtuins) have been demonstrated. In addition, both AHR and CD38 are constituents of a signalsome of neutrophils (Bunaciu et al. 2019) that is probably involved in metabolic adaptation between inflammation and associated energy demands.

Roles of AHR and NAD^+^-consuming enzymes in tissue-dependent inflammatory diseases have been discussed using two examples: infectious colitis and obesity-mediated NAFLD and NASH. In intestinal inflammation, beneficial effects have been discussed in homeostasis, microbial defense and the resolution phase of inflammation, provided that AHR is transiently activated. In NASH, both AHR and CD38 may be involved in anti-inflammatory processes. Therefore, both AHR-activating and CD38-inhibiting phytochemicals and microbial products as well as NAD^+^ boosting compounds may provide therapeutic options. However, anti-inflammatory tolerance mechanisms bear the risk to facilitate autoimmune diseases and carcinogenesis. Therefore, clinical studies have to evaluate risks and benefits in particular cases. The discussed perceptions may be applicable to inflammatory diseases of other organs including skin and lung.

TCDD toxicity is known to be species dependent leading to wasting syndrome and lethality in sensitive species (Poland and Knutson 1982). TCDD toxicity models in sensitive species, in particular in the chick embryo model, provide valuable hints to molecular AHR targets of energy metabolism. The results point to NAD^+^-dependent nuclear SIRT1 and mitochondrial SIRT3 as key regulators of gluconeogenesis and glycolytic enzymes. Hence, AHR research remains a challenging and promising field.
